# Multicenter evaluation of an automated, multiplex, RNA-based molecular assay for detection of *ALK*, *ROS1*, *RET* fusions and *MET* exon 14 skipping in NSCLC

**DOI:** 10.1007/s00428-024-03778-9

**Published:** 2024-03-16

**Authors:** Linea Melchior, Astrid Hirschmann, Paul Hofman, Christophe Bontoux, Angel Concha, Salima Mrabet-Dahbi, Pascal Vannuffel, Emmanuel Watkin, Martina Putzová, Stefania Scarpino, Anne Cayre, Paloma Martin, Robert Stoehr, Arndt Hartmann

**Affiliations:** 1 Department of Pathology, Rigshospitalet, University Hospital of Copenhagen, Copenhagen, Denmark; 2 Copenhagen, Denmark; 3 Department of Pathology, Luzerner Kantonsspital, Lucerne, Switzerland; 4 Laboratory of Clinical and Experimental Pathology, Hôpital Pasteur, Centre Hospitalier Universitaire de Nice, Université Côte d’Azur, Nice, France; 5 Hospital-integrated Biobank (BB-0033-00025), Hôpital Pasteur, Nice, France; 6 FHU OncoAge, IHU RespirERA, Hôpital Pasteur, Centre Hospitalier Universitaire de Nice, Université Côte d’Azur, Nice, France; 7 Complejo Hospitalario de A Coruña, Corunna, Spain; 8 Institut für Pathologie, Klinikum Kassel, Kassel, Germany; 9 Institut de Pathologie et de Génétique, Gosselies, Belgium; 10 Cabinet de Pathologie Cypath, Lyon, France; 11 Bioptická Laboratoř, Pilsen, Czechia; 12 Department of Clinical and Molecular Medicine, Pathology Unit, St. Andrea University Hospital, University of Rome La Sapienza, Rome, Italy; 13 UF de Pathologie, Centre Jean Perrin, INSERM U1240, Clermont-Ferrand, France; 14 Molecular Pathology Group, Department of Pathology, Instituto de Investigación Sanitaria Puerta de Hierro-Segovia de Arana (IDIPHISA), Madrid, Spain; 15 Centro de Investigación Biomédica en Red de Cáncer (CIBERONC), Madrid, Spain; 16 Institute of Pathology, University Erlangen-Nürnberg, Erlangen, Germany; 17 Comprehensive Cancer Center Erlangen EMN, Erlangen, Germany; 18 Bavarian Cancer Research Center (BZKF), Erlangen, Germany

**Keywords:** Idylla, NSCLC, *ALK* fusion, *ROS1* fusion, *RET* fusion, *MET* exon 14 skipping

## Abstract

The current study assessed the performance of the fully automated RT-PCR-based Idylla™ GeneFusion Assay, which simultaneously covers the advanced non-small cell lung carcinoma (aNSCLC) actionable *ALK*, *ROS1*, *RET*, and *MET* exon 14 rearrangements, in a routine clinical setting involving 12 European clinical centers. The Idylla™ GeneFusion Assay detects fusions using fusion-specific as well as expression imbalance detection, the latter enabling detection of uncommon fusions not covered by fusion-specific assays. In total, 326 archival aNSCLC formalin-fixed paraffin-embedded (FFPE) samples were included of which 44% were resected specimen, 46% tissue biopsies, and 9% cytological specimen. With a total of 179 biomarker-positive cases (i.e., 85 *ALK*, 33 *ROS1*, 20 *RET* fusions and 41 *MET* exon 14 skipping), this is one of the largest fusion-positive datasets ever tested. The results of the Idylla™ GeneFusion Assay were compared with earlier results of routine reference technologies including fluorescence in situ hybridization, immunohistochemistry, reverse-transcription polymerase chain reaction, and next-generation sequencing, establishing a high sensitivity/specificity of 96.1%/99.6% for *ALK*, 96.7%/99.0% for *ROS1*, 100%/99.3% for *RET* fusion, and 92.5%/99.6% for *MET* exon 14 skipping, and a low failure rate (0.9%). The Idylla™ GeneFusion Assay was found to be a reliable, sensitive, and specific tool for routine detection of *ALK*, *ROS1*, *RET* fusions and *MET* exon 14 skipping. Given its short turnaround time of about 3 h, it is a time-efficient upfront screening tool in FFPE samples, supporting rapid clinical decision making. Moreover, expression-imbalance-based detection of potentially novel fusions may be easily verified with other routine technologies without delaying treatment initiation.

**Supplementary Information:**

The online version contains supplementary material available at 10.1007/s00428-024-03778-9.

## Introduction

Non-small cell lung carcinoma (NSCLC) therapy has been transformed by the identification of actionable oncogenic driver mutations, of which *ALK*, *ROS1*, *RET* fusions and *MET* exon 14 skipping represent interesting drug targets [1]. ALK [2], ROS1 [3], and RET [4] are tyrosine kinases, and fusion of their kinase domains with the amino-terminal portions of a variety of protein partners leads to disturbance of downstream signaling cascades, resulting in uncontrolled cell proliferation and promotion of survival. *MET* exon 14 skipping on the other hand is an intragenic rearrangement of the *MET* gene resulting in sustained MET activation, ultimately leading to cell proliferation and tumor growth [5]. The prevalence of *ALK* fusion events in NSCLC is 4–7%, for *ROS1* prevalence is 1–3%, and for *RET* it is 1–2% [6–8], while *MET* exon 14 skipping has a prevalence of 3% [8]. Therefore, the combined frequency of these gene rearrangements is about the same as the occurrence of *EGFR* mutations in an European advanced NSCLC population [9].

To determine eligibility to therapy targeting *ALK*, *ROS1*, *RET*, or *MET* alterations [10–13], NSCLC treatment guidelines recommend testing for *ALK*, *ROS1*, *RET* fusions and *MET* exon 14 skipping [14, 15]. Kinase fusions are usually detected using single-test methods like immunohistochemistry (IHC) for *ALK* fusions [15–17], and fluorescence in situ hybridization (FISH), which is the gold standard for detection of *ALK* and *ROS1* fusions and to a lesser extent *RET* fusions [18, 19], or multiplex test methods like reverse-transcription polymerase chain reaction (RT-PCR) and next-generation sequencing (NGS) [20] as valuable alternatives to *ALK*, *ROS1*, and *RET* FISH and as the most effective detection methods for *MET* exon 14 skipping.

The CE-IVD Idylla™ GeneFusion Assay (Biocartis, Mechelen, Belgium) is an RNA-based fully automated RT-PCR assay, designed to concurrently detect presence of *ALK*, *ROS1*, *RET* fusions and *MET* exon 14 skipping in formalin-fixed, paraffin-embedded (FFPE) tumor tissue sections. This is realized by combining (i) specific detection of *ALK*, *ROS1*, *RET* fusions and *MET* exon 14 skipping and (ii) analysis of *ALK*, *ROS1*, and *RET* expression imbalance. The latter measures the difference between 3′ and 5′ gene expression levels of *ALK*, *ROS1*, or *RET*, which is indicative for the presence of a fusion, however, should always be confirmed with an alternative gene fusion test method [21]. The research which use only (RUO) version of the Idylla™ GeneFusion Assay, which also covered *NTRK1*/*2*/*3* rearrangements, was launched before the CE-IVD Idylla™ GeneFusion Panel. In initial studies, this RUO Idylla™ GeneFusion Assay showed high sensitivity (82–100%) and specificity (98–100%) for the detection of *ALK*, *ROS1*, *RET* fusions and *MET* exon 14 skipping [22, 23]. In a recently published study, the Idylla™ GeneFusion Assay had an equal level of gene fusion detection compared with the Genexus NGS system, which has a turnaround time (not including RNA purification) of about 24 h; with each ultrafast gene fusion assay having its own specific technology-related limitations [24]. Due to sample-to-result automatization and only 2 min hands-on time required to load tissue slides into the single-use cartridge, the Idylla™ GeneFusion Assay can be easily established in on-site routine screening. Together with the absence of batching need and its short turnaround time of approximately 3 h, the technology may as such address the relatively long turnaround times associated with NGS [25–29].

The current multicenter study investigated the performance of the Idylla™ GeneFusion Assay (RUO) in a real-life clinical setting involving 12 clinical centers across Europe. These centers selected and tested a total of 326 archival histologically proven advanced NSCLC (stage IV) FFPE tissue samples with the Idylla™ GeneFusion Assay of which results were compared with the biomarker status determined earlier with routine reference methods (including FISH, IHC, RT-PCR, and NGS).

## Materials and methods

### Tissue sample collection and study design

Twelve clinical centers from nine different European countries participated in this multicenter observational non-interventional retrospective study that assessed the mutational status of 326 archival, histologically proven, advanced NSCLC (stage IV) tissue or cytological FFPE samples (Table 1). All samples had been tested previously with a routine reference method for *ALK*, *ROS1*, *RET* fusions and/or *MET* exon 14 skipping, and the current study retested them at the same clinical centers using the Idylla™ GeneFusion Assay.

**Table 1:** Participating clinical centers.

	Laboratory	City	Country	Number of samples
1	Institut de Pathologie et de Génétique	Gosselies	Belgium	13
2	Bioptická Laboratoř	Pilsen	Czech Republic	29
3	Rigshospitalet	Copenhagen	Denmark	29
4	Laboratory of Clinical and Experimental Pathology, CHU Nice	Nice	France	30
5	Cypath	Lyon	France	27
6	Centre Jean-Perrin	Clermont-Ferrand	France	32
7	Universitätsklinik Erlangen	Erlangen	Germany	30
8	Klinikum Kassel	Kassel	Germany	10
9	Sant Andrea Roma	Rome	Italy	28
10	Complejo Hospitalario de A Coruña	Corunna	Spain	31
11	Hospital Universitario Puerta de Hierro-Majadahonda	Madrid	Spain	30
12	Luzerner Kantonsspital	Lucerne	Switzerland	37
	Total			326

Patients provided informed consent. The use of these patient samples was approved by the respective local Ethics Committees and was in accordance with the Declaration of Helsinki.

For inclusion, each sample had to originate from the same block that was used with the original reference method test and was required to have ≥10% neoplastic cells and a tissue area >20 mm^2^ when 1 slice/slide was used, or a tissue area ≤20 mm^2^ if three slices/slides were used. FFPE cell block requirements were ≥10% neoplastic cells and total cell number >2000 per slide when 1 slice/slide was used or neoplastic cell number ≤2000 per slide when three slices/slides were used. Stained samples, non-FFPE samples, decalcified samples, and samples older than 9 years (or 3 years if compared to FISH) were excluded. Macrodissection was allowed to increase neoplastic cell content to above the required 10%.

Sampling of FFPE tissue sections (mostly one or two, up to five slides) for the Idylla™ GeneFusion Assay was performed consecutively, if possible, to the sections used in the earlier routine reference method. For each sample, the tissue section(s) were placed in the lysis chamber of a new cartridge, which was next loaded onto the Idylla™ instrument. The whole procedure required about 2 min of hands-on time.

The results obtained with the Idylla™ GeneFusion Assay on archival material were not used for diagnostic purposes.

### Idylla™ GeneFusion Assay

The real-time PCR-based Idylla™ GeneFusion Assay (RUO) was used in the current study. This is a fully automated in vitro diagnostic test qualitatively detecting specific *ALK*, *ROS1*, *RET* gene fusions as well as *MET* exon 14 skipping from RNA transcripts. The Idylla™ GeneFusion Assay gene panel is detailed in Supplementary Table 1. Apart from the detection of these specific fusions, the Idylla™ GeneFusion Assay (RUO) does also assess *ALK*, *ROS1*, *RET*, and *NRTK1/2/3* expression imbalance. *NRTK1/2/3* expression imbalance results are not reported here as *NRTK1/2/3* fusion detection was not in the scope of the current study.

The Idylla™ GeneFusion Assay covers the entire sample-to-result process, including fully integrated RNA extraction (which is based on a combination of enzymatic degradation, heat, and high-frequency ultrasound), reverse transcription of RNA to cDNA, real-time PCR amplification and detection, as well as data analysis, and result reporting.

### Routine methods used as reference method

Several commercial FISH assays were used as reference method following the manufacturer’s instructions: Abbot (Abbott, Abbott Park, IL) Vysis break apart probe (*ALK* fusion), Leica (Wetzlar, Germany) XL for BOND, ZytoVision (Bremerhaven, Germany) Zytolight SPEC Dual Color break apart probe (*ALK*, *ROS1*, *RET* fusions), and Empire Genomics (Buffalo, NY; *ALK*, *ROS1*, *RET* fusions).

The following commercial IHC methods were used according to the manufacturer’s instructions: Roche Diagnostics (Rotkreuz, Switzerland) Ventana D5F3 (*ALK* fusion), Ventana SP384 (*ROS1* fusion), Abcam (Waltham, MA) 5A4 (*ALK* fusion), Cell Signaling (Danvers, MA) D4D6 (*ROS1* fusion), Bond Leica D5F3 (*ALK* fusion), and Leica Bond D4D6 (*ROS1* fusion).

Routine NGS methods that were used according to the manufacturer’s instructions were Archer (Boulder, CO) Fusionplex Lung v1.0 (*ALK*, *ROS1*, *RET* fusions, *MET* exon 14 skipping), Thermo Fisher Scientific (Waltham, MA) Oncomine Focus Assay (*ALK*, *ROS1*, *RET* fusions, *MET* exon 14 skipping), Thermo Fisher Scientific Oncomine Precision Assay (*RET* fusion, *MET* exon 14 skipping), and Illumina (San Diego, CA) TruSight Tumor 170 (*ALK*, *ROS1*, *RET* fusions, *MET* exon 14 skipping).

The Diatech (Jesi, Italy) Easy PGX RT025 (*ALK*, *ROS1*, *RET* fusions, *MET* exon 14 skipping) and AmoyDx (Xiamen, China) Pan Lung Cancer panel (*ALK* fusion, *MET* exon 14 skipping) RT-PCR assays were used following the manufacturer’s instructions.

A Sanger assay was used to determine *MET* exon 14 skipping.

### Statistical analysis

Concordance was calculated using overall concordance, sensitivity, and specificity, excluding invalid and error test results. Failure rate was calculated as the sum of errors and invalid test results on the total sample set.

## Results

### Sample description

The current study evaluated the Idylla™ GeneFusion Assay in a real-life clinical setting. To this end, a set of 326 archival, unstained FFPE advanced NSCLC (stage IV) tissue or cytological samples was selected by 12 centers across nine European countries. For two of these samples, the Idylla™ GeneFusion Assay resulted in an error, and therefore the final analysis set contained 324 samples of which the characteristics are summarized in Table 2. All samples had been tested before at these clinical centers to detect *ALK*, *ROS1*, *RET* fusions and/or *MET* exon 14 skipping using the previously described range of validated reference methods, and the current study assesses the concordance between the Idylla™ GeneFusion Assay results and results of these former routine reference methods. Of the 324 samples tested, 179 (55%) had been reported by the routine reference methods as positive for *ALK*, *ROS1*, *RET* fusions and/or for *MET* exon 14 skipping, and 145 (45%) had been reported as negative for these gene alterations.

**Table 2:** Sample characteristics (*n*=324)

Characteristic			*n*	%
Sample size	NSCLC		324	**100%**
Sample type, FFPE	Biopsy		150	**46%**
	Resection		144	**44%**
	Cytological		28	**9%**
	Unknown		2	**1%**
Origin of tissue	Primary		217	**67%**
		Lung	214	99%
		Lymph node	1	0.5%
		Not known	2	1%
	Metastatic		94	**29%**
		Lung	25	27%
		Lymph node	36	39%
		Brain	6	6%
		Liver	4	4%
		Adrenal gland	1	1%
		Skin	1	1%
		Other	20	21%
		Not known	1	1%
	Unknown		13	**4%**
Reference methods	*ALK* fusion		85	**26%**
		IHC	15	18%
		FISH	16	19%
		NGS	23	27%
		PCR	1	1%
		IHC/FISH	13	15%
		IHC/NGS	5	6%
		FISH/NGS	3	4%
		IHC/FISH/NGS	7	8%
		IHC/FISH/PCR	2	2%
	*ROS1* fusion		33	**10%**
		IHC	6	18%
		FISH	4	12%
		NGS	11	33%
		IHC/FISH	4	12%
		FISH/NGS	2	6%
		IHC/FISH/NGS	6	18%
	*RET* fusion		20	**6%**
		FISH	7	35%
		NGS	12	60%
		AmoyDx	1	5%
	*MET* exon 14 skipping		41	**13%**
		NGS	27	66%
		PCR	5	12%
		NGS/PCR	9	22%

*FFPE* formalin-fixed paraffin-embedded, *FISH* fluorescence in situ hybridization, *IHC* immunohistochemistry, *NGS* next-generation sequencing, *NSCLC* non-small-cell lung carcinoma, *PCR* polymerase chain reaction. Bold: Frequency of different input materials, frequency of primary vs. metastatic NSCLC and frequency of fusions in the cohort

Considering the neoplastic cell content, six samples (2%) deviated from the protocol instructions to have at least 10% neoplastic cell content, and for four samples neoplastic cell content was not recorded. About two-thirds (69%) of the slides tested had a thickness of 5 μm, while one-third (30%) had a thickness of 10 μm; in five cases, slide thickness was not reported. Most often one (30%) or two (37%) slides were tested; for 14 samples, the number of slides tested was not reported. The tissue surface varied from below 20 mm^2^ (19%), to between 20 and 50 mm^2^ (36%), between 50 and 100 mm^2^ (14%), and above 100 mm^2^ (20%), while tissue surface was not reported in 38 cases. Macrodissection to increase neoplastic cell content was performed on 156 samples (48%).

Overall, the sample set offered a large sample size, included several clinical routine workflows, and was considered representative for real-life clinical circumstances.

### Results of Idylla™ GeneFusion Assay compared with reference methods

Slides of 324 archival samples tested with the Idylla™ GeneFusion Assay obtained 323 valid overall test results, which were compared with the results of the routine reference methods that were previously performed on the same tissue block (Table 3). One invalid test result was obtained using the Idylla™ GeneFusion Assay, for which the reference reported an *ALK* fusion; the sample tested was however smaller (0–20 mm^2^) with low tumor cell content (10–20%) and confirmed to be a less-quality sample as commented to be indicative for RNA degradation.

**Table 3:** Comparison between results of the Idylla™ GeneFusion Assay and routine reference methods for the detection of *ALK*, *ROS1*, *RET* fusions and *MET* exon 14 skipping

Idylla™ GeneFusion Assay	Reference method			Reference method		
	Detected	Non-detected	Total	Detected	Non-detected	Total
	*ALK* fusion results only			*ALK* fusion including expression imbalance		
Detected	66	1	67	74	1	75
Non-detected	18	238	256	10	238	248
Invalid	1	0	1	1	0	1
Total	85	239	324	85	239	324
	*ROS1* fusion results only			*ROS1* fusion including expression imbalance		
Detected	25	3	28	28	3	31
Non-detected	8	287	295	5	287	292
Invalid	0	1	1	0	1	1
Total	33	291	324	33	291	324
	*RET* fusion results only			*RET* fusion including expression imbalance		
Detected	15	1	16	19	1	20
Non-detected	5	302	307	1	302	303
Invalid	0	1	1	0	1	1
Total	20	304	324	20	304	324
	*MET* exon 14 skipping					
Detected	37	0	37			
Non-detected	4	282	286			
Invalid	0	1	1			
Total	41	283	324			

A first comparison of the data showed that the Idylla™ GeneFusion Assay reported one *ALK* fusion, three *ROS1* fusions, and one *RET* fusion not detected by the routine reference method. Inversely, the Idylla™ GeneFusion Assay did not confirm 18 *ALK* fusions, eight *ROS1* fusions, five *RET* fusions, and four *MET* exon 14 skipping events previously reported by routine reference methods.

In addition to the different particular fusion events, the Idylla™ GeneFusion assay also measures *ALK*, *ROS1*, and *RET* expression imbalance, which could be indicative for the presence of a fusion. When including in the analysis of the expression imbalance results, the Idylla™ GeneFusion Assay detected eight additional *ALK* fusion events, three *ROS1* fusions, and four *RET* fusions.

Including the expression imbalance results, there was an agreement between the Idylla™ GeneFusion Assay and the routine reference methods for 312 (96.3%) samples regarding *ALK* fusion, 315 (97.2%) samples for *ROS1* fusion, 321 (99.1%) samples for *RET* fusion, and 319 (98.5%) samples for *MET* exon 14 skipping. The samples with discordant results were further analyzed.

### Analysis of discordant results

The samples for which the results of the Idylla™ GeneFusion Assay and the reference method were discordant and for which enough sample material was still available, were re-analyzed using an additional test on material from the same FFPE tissue block. The additional test method is indicated in Table 4, together with its results. The “true value” is considered the value confirmed by at least two different technologies. If the re-analyzed result or third-method test result confirmed the Idylla™ GeneFusion Assay result, the outcome was reclassified as concordant.

**Table 4:** Retest results for samples discordant between the Idylla™ GeneFusion Assay (including expression imbalance results for *ALK*, *ROS1*, and *RET* fusions) and the previously used routine reference method(s)

Sample	Idylla™	FISH	IHC	NGS/PCR	Additional method	True value	Conclusion
*ALK* fusion							
1	−	na	−	+		Not detected	Concordant
2	−	na	+	−		Not detected	Concordant
3	−	na	na	+	Second NGS	Not detected	Concordant
4	−	+	-	na	NGS	Not detected	Concordant
5	−	+	na	na	NGS	Not detected	Concordant
6	−	+	na	na	NGS	Not detected	Concordant
7	−	+	na	na	NGS	Not detected	Concordant
8	−	na	+	na	NGS	Detected	Discordant
9	−	na	na	+	FISH	Not detected	Concordant
10	−	+	+	na		Detected	Discordant
11	+	−	−	na		Not detected	Discordant
*ROS1* fusion							
12	−	−	+	−		Not detected	Concordant
13	−	−	+	−		Not detected	Concordant
14	−	na	+	−		Not detected	Concordant
15	−	−	+	na		Not detected	Concordant
16	−	na	na	+	No third method	na	Inconclusive
17	+	na	−	−		Not detected	Discordant
18	+	na	−	−		Not detected	Discordant
19	+	na	na	−	No third method^^^	Detected	Concordant
*RET* fusion							
20	−	+	na	−		Not detected	Concordant
21	+	na	na	na	No third method*	Not detected	Discordant
*MET* exon 14 skipping							
22	−	na	na	+	Second NGS	Detected	Discordant
23	−	na	na	+	No third method	na	Inconclusive
24	−	na	na	+	No third method	na	Inconclusive
25	−	na	na	+	No third method^#^	Not detected	Concordant

*FISH* fluorescence in situ hybridization, *IHC* immunohistochemistry, *NGS* next-generation sequencing, *na* not applicable, *PCR* polymerase chain reaction. ^Repeat data analysis original NGS. *Idylla™ software error, resolved with new release. ^#^Reference method (Sanger) error

For sample 3, a second NGS assay confirmed the absence of *ALK* fusion and therefore confirmed the Idylla™ GeneFusion Assay result. As part of the analysis of discordant samples, samples 4 and 9 were retested at the original study center with NGS and FISH, respectively, both resulting in a non-detected *ALK* fusion result. Both cases were therefore classified as “not detected” for “true value” and concluded to be concordant. For samples 5, 6, 7, and 8, a third-method analysis was performed at an independent testing site using NGS. In three cases (samples 5, 6, 7), NGS resulted in a non-detected *ALK* fusion result confirming false positive results with FISH, while in one case (sample 8), it confirmed the IHC result and hence a false negative result with the Idylla™ GeneFusion Assay. Sample 11 was repeated twice with the Idylla™ GeneFusion Assay at the original study center, resulting in two positive *ALK* and one negative *ALK* detection overall. It was decided to label this sample as discordant. For samples 18 and 19, two biomarkers were detected with the Idylla™ GeneFusion Assay, i.e., *ROS1* fusion + *MET* exon 14 skipping for sample 18 and *ROS1* fusion + *ALK* fusion for sample 19. *MET* exon 14 skipping (sample 18) and *ALK* fusion (sample 19) were detected by NGS as well. However in both cases, a *ROS1* fusion had been detected at very low read count by the Archer NGS panel used at the original study center but had not been reported. For sample 19, this *ROS1* rearrangement involved an intergenic region as well, and based on a profound re-analysis of the Archer NGS results, it was decided to label this sample as concordant (Supplementary Figure 1). In sample 21, the Idylla™ GeneFusion Assay detected a double fusion (i.e., *ALK* fusion + *RET* fusion), of which the *RET* fusion was not reported by the reference methods as they did not test for this rearrangement. However, further analysis revealed that this detection of a *RET* fusion by the Idylla™ GeneFusion Assay was due to a software error, which the manufacturer planned to correct with a software update. Third-method testing of sample 22 with a second NGS test (i.e., Oncomine Focus Assay), this time at an independent testing site, confirmed the presence of *MET* exon 14 skipping (read count of 397 reads). For sample 25, the site confirmed that the initial *MET* exon 14 skipping detection with Sanger was not a true *MET* exon 14 skipping variant, and therefore the “true value” was classified as “not detected.” Sample 16, 23, and 24 did not have sufficient sample available for a discordant analysis with an additional method. For sample 16, a *CCDC6*::*ROS1* fusion was detected with NGS; CCDC6 is a partner gene not included in the Idylla™ GeneFusion Assay fusion-specific design.

### Performance of Idylla™ GeneFusion Assay

Based on the discordant analysis results, eight additional samples were classified as *ALK* fusion concordant, as well as five additional samples for *ROS1* fusion, one additional sample for *RET* fusion, and one additional sample for *MET* exon 14 skipping (Table 5). The resulting overall concordance in this dataset of the Idylla™ GeneFusion Assay (including expression imbalance) with reference method results was 98.8% for *ALK* fusion, 98.8% for *ROS1* fusion, 99.4% for *RET* fusion, and 98.8% for *MET* exon 14 skipping. The inconclusive cases were considered to be discordant for the final calculation. Given the three failures reported above (i.e., two errors and one invalid result), the validity of the Idylla™ GeneFusion Assay was 99.1% (323/326).

**Table 5:** Comparison between results of the Idylla™ GeneFusion Assay and routine reference methods for the detection of *ALK*, *ROS1*, *RET* fusions and *MET* exon 14 skipping after further analysis of discordant results

Idylla™ GeneFusion Assay	Reference method			Sensitivity	Specificity	Overall concordance
	Detected	Non-detected	Total			
*ALK* fusion including expression imbalance						
Detected	74	1	75			
Non-detected	2	246	248			
Invalid	1	0	1			
Total	77	247	324	96.1% (74/77)	99.6% (246/247)	98.8% (320/324)
*ROS1* fusion including expression imbalance						
Detected	29	2	31			
Non-detected	1	291	292			
Invalid	0	1	1			
Total	30	294	324	96.7% (29/30)	99.0% (291/294)	98.8% (320/324)
*RET* fusion including expression imbalance						
Detected	19	1	20			
Non-detected	0	303	303			
Invalid	0	1	1			
Total	19	305	324	100% (19/19)	99.3% (303/305)	99.4% (322/324)
*MET* exon 14 skipping						
Detected	37	0	37			
Non-detected	3	283	286			
Invalid	0	1	1			
Total	40	284	324	92.5% (37/40)	99.6% (283/284)	98.8% (320/324)

## Discussion

In patients with advanced NSCLC with rapid disease progression, timely therapeutic decision making is essential. In the past decade, treatment options for NSCLC have expanded, which led to an increased number of biomarkers to be tested, often on very sparse material with multiple testing technologies. This may result in an undesired prolonged time to treatment for this vulnerable group of patients.

The current multicenter study investigated the performance of the Idylla™ GeneFusion Assay in a real-life clinical setting involving 12 clinical centers across Europe. These centers selected and tested a total of 326 archival histologically proven advanced NSCLC (stage IV) FFPE tissue samples with the Idylla™ GeneFusion Assay of which results were compared with the molecular status determined earlier with routine reference methods including FISH, IHC, RT-PCR, and NGS.

Among the 326 samples analyzed, a very low failure rate of 0.9% (3/326) was observed for the Idylla™ GeneFusion Assay. This despite the analysis been carried out on archival material with up to 9 years between the initial analysis and the current study, the included material originating from various metastatic sites with different pre-analytic tissue preparation procedures, and the analysis being carried out at 12 different sites with different tissue processing and storage procedures. This observation supports the robustness of the assay as a fast and reliable test in a real-life diagnostic setting.

The 324 remaining samples tested comprised of 179 (55%) samples reported as positive for either *ALK*, *ROS1*, *RET* fusions and/or *MET* exon 14 skipping, and 145 (45%) samples reported as negative for these gene alterations by the routine reference method, providing a large sample size to assess both sensitivity and specificity of the Idylla™ GeneFusion Assay, with different tissues and different fusion compositions, but also offering a unique opportunity to test it against the routine reference methods currently used in routine NSCLC molecular testing.

It was found that the Idylla™ GeneFusion Assay has a high sensitivity/specificity, respectively, of 96.1%/99.6% for *ALK*, 96.7%/99.0% for *ROS1*, 100%/99.3% for *RET* fusions and 92.5%/99.6% for *MET* exon 14 skipping. It was clearly demonstrated that the expression imbalance technology has its value in increasing the sensitivity of the assay and thereby acts as the complement for detection of fusion transcripts with uncommon or novel fusion partners in NSCLC, especially in the case of *ALK* and *RET* fusions. In this study, only expression-imbalance-positive results that were confirmed by an alternative method were considered true positive, as per the manufacturer’s assay instructions. This makes the Idylla™ GeneFusion Assay expression imbalance technology highly relevant as a time-efficient upfront screening tool where detected imbalances can be verified quickly with either IHC or FISH, and the relatively slower NGS can be performed afterward to establish the exact fusion present, without delaying treatment initiation. In a recent investigation conducted by Gilson *et al.* in 2023, diverse potential applications of the Idylla™ system within laboratory workflows have been elucidated. These applications encompass integration in the form of a fast-track precursor to comprehensive testing, a sequential approach, or as a rescue test. It is imperative for each institution to conduct a thorough assessment of its unique clinical requirements and available resources, taking into consideration logistical and financial parameters, in order to determine the most suitable integration strategy [30]. The Idylla™ GeneFusion Assay can be used on demand without the need of batching, directly starting from limited FFPE material and with results available within 3 h.

These findings make the Idylla™ GeneFusion Assay an obvious choice for fast and reliable detection of treatment-relevant fusions in the initial NSCLC diagnostic workup. Fast detection of targetable fusions may however also be highly relevant for NSCLC patients progressing on current tyrosine kinase inhibitor treatment, as oncogenic fusions are one of many known tyrosine kinase inhibitor resistance mechanisms [31, 32].

As with all retrospective studies, one of the study limitations is the retrospective design and hence the sample selection bias. In an ideal setting, all the included samples would have been tested with several or all reference methods considered but given the limited sample availability and the diversity in methods applied at the different sites, like in a real-life setting, this was not an option.

The study design tried to ensure that only samples with enough material available to perform an additional in-depth discordant investigation were included, but this was unfortunately not the case for three of the 25 discordant samples. It can be quite difficult to assess the amount of available material left in a FFPE tissue block containing small lung biopsies or cell block material, and the availability can be further limited for fusion-positive samples if additional analysis has been performed as part of the initial diagnostic workup. One could of course exclude these samples from the study, but as they reflect the limitations of a retrospective study and the normal challenges faced in a thoracic oncology testing facility, they were included.

The same rationale was behind inclusion of the inconclusive cases in the final calculations of sensitivity and specificity for detection of *ALK*, *ROS1*, *RET* fusions and *MET* exon 14 skipping. They represent a real-life clinical cohort, and the result obtained with the reference method was therefore considered the “true value” if a third method could not be performed or its result was inconclusive. Retrospective studies can have difficulties to fully elucidate the real “true value” due to limited material and resources.

The true value for the discordant *MET* exon 14 skipping cases may be hard to establish if the reference or additional NGS method used is the Oncomine technology, which can create false positive calls, due to a homopolymeric error of the splice donor site [33]. These false positive calls can be distinguished by relatively low read counts compared to real *MET* exon 14 skipping events. The two inconclusive *MET* exon 14 skipping cases may reflect this pitfall.

To conclude, the Idylla™ GeneFusion Assay is a promising tool for rapid detection of *ALK*, *ROS1*, *RET*, or *MET* exon 14 alterations in NSCLC.

### Supplementary information

ESM 1 Supplementary Table 1: *ALK*, *ROS1*, and *RET* fusions and *MET* exon 14 skipping mutations included in the panel of the Idylla™ GeneFusion Assay. (DOCX 35 kb)

ESM 2 Supplementary Figure 1: Sample 19 Archer NGS routine reference result. (DOCX 1900 kb)

## Data Availability

The datasets generated during and/or analyzed during the current study are available from the corresponding author on reasonable request.
