# Correction to: Multicenter evaluation of an automated, multiplex, RNA-based molecular assay for detection of ALK, ROS1, RET fusions and MET exon 14 skipping in NSCLC

**DOI:** 10.1007/s00428-024-03800-0

**Published:** 2024-04-19

**Authors:** Linea Melchior, Astrid Hirschmann, Paul Hofman, Christophe Bontoux, Angel Concha, Salima Mrabet‑Dahbi, Pascal Vannuffel, Emmanuel Watkin, Martina Putzová, Stefania Scarpino, Anne Cayre, Paloma Martin, Robert Stoehr, Arndt Hartmann

**Affiliations:** 1 Department of Pathology, Rigshospitalet, University Hospital of Copenhagen, Copenhagen, Denmark; 2 Copenhagen, Denmark; 3 Department of Pathology, Luzerner Kantonsspital, Lucerne, Switzerland; 4 Laboratory of Clinical and Experimental Pathology, Hôpital Pasteur, Centre Hospitalier Universitaire de Nice, Université Côte d’Azur, Nice, France; 5 Hospital-integrated Biobank (BB, Hôpital Pasteur, 0033-00025 Nice, France; 6 FHU OncoAge, IHU RespirERA, Hôpital Pasteur, Centre Hospitalier Universitaire de Nice, Université Côte d’Azur, Nice, France; 7 Complejo Hospitalario de A Coruña, Corunna, Spain; 8 Institut für Pathologie, Klinikum Kassel, Kassel, Germany; 9 Institut de Pathologie et de Génétique, Gosselies, Belgium; 10 Cabinet de Pathologie Cypath, Lyon, France; 11 Bioptická Laboratoř, Pilsen, Czechia; 12 Department of Clinical and Molecular Medicine, Pathology Unit, St. Andrea University Hospital, University of Rome La Sapienza, Rome, Italy; 13 UF de Pathologie, Centre Jean Perrin, INSERM U1240, Clermont-Ferrand, France; 14 Molecular Pathology Group, Department of Pathology, Instituto de Investigación Sanitaria Puerta de Hierro-Segovia de Arana (IDIPHISA), Madrid, Spain; 15 Centro de Investigación Biomédica en Red de Cáncer (CIBERONC), Madrid, Spain; 16 Institute of Pathology, University Erlangen-Nürnberg, Erlangen, Germany; 17 Comprehensive Cancer Center Erlangen EMN, Erlangen, Germany; 18 Bavarian Cancer Research Center (BZKF), Erlangen, Germany

**Correction to: Virchows Archiv**

10.1007/s00428-024-03778-9

In the original version of this article, the given and family names of the authors were interchanged. The names were displayed correctly in all versions at the time of publication.

The original article has been corrected.

