# Supplementary material for: Multicenter evaluation of an automated, multiplex, RNA-based molecular assay for detection of ALK, ROS1, RET fusions and MET exon 14 skipping in NSCLC
Source: Virchows Arch. 2024 Mar 16;484(4):677–86. doi: 10.1007/s00428-024-03778-9 (PMC11062995; doi:10.1007/s00428-024-03778-9)
Supplement: Supplementary file 1 — Supplementary Table 1: ALK, ROS1, and RET fusions and MET exon 14 skipping mutations included in the panel of the Idylla™ GeneFusion Assay. (DOCX 35 kb) [file 428_2024_3778_MOESM1_ESM.docx]

Supplementary Table 1: *ALK*, *ROS1*, and *RET* fusions and *MET* exon 14 skipping mutations included in the panel of the Idylla™ GeneFusion Assay.

| **Fusion name** | **Exons involved** |
| --- | --- |
| ***ALK* fusions (17)** | |
| EML4-ALK | *EML4* exon 2; *ALK* exon 20 |
|  | *EML4* exon 6a; *ALK* exon 20 |
|  | *EML4* exon 6b; *ALK* exon 20 |
|  | *EML4* exon 13; *ALK* exon 20 |
|  | *EML4* exon 15; *ALK* exon 20 |
|  | *EML4* exon 17; *ALK* exon 20 |
|  | *EML4* exon 18; *ALK* exon 20 |
|  | *EML4* exon 20; *ALK* exon 20 |
| KIF5B-ALK | *KIF5B* exon 15; *ALK* exon 20 |
|  | *KIF5B* exon 17; *ALK* exon 20 |
|  | *KIF5B* exon 24; *ALK* exon 20 |
| HIP1-ALK | *HIP1* exon 28; *ALK* exon 20 |
|  | *HIP1* exon 30; *ALK* exon 20 |
| KLC1-ALK | *KLC1* exon 9; *ALK* exon 20 |
| TPR-ALK | *TPR* exon 15; *ALK* exon 20 |
| TFG-ALK | *TFG* exon 4; *ALK* exon 20 |
|  | *TFG* exon 6; *ALK* exon 20 |
| ***ROS1* fusions (13)** | |
| CD74-ROS1 | *CD74* exon 6; *ROS1* exon 32 |
|  | *CD74* exon 6; *ROS1* exon 34 |
| SDC4-ROS1 | *SDC4* exon 2; *ROS1* exon 32 |
|  | *SDC4* exon 4; *ROS1* exon 32 |
|  | *SDC4* exon 4; *ROS1* exon 34 |
| SLC34A2-ROS1 | *SLC34A2* exon 4; *ROS1* exon 32 |
|  | *SLC34A2* exon 4; *ROS1* exon 34 |
|  | *SLC34A2* exon 13; *ROS1* exon 32 |
| EZR-ROS1 | *EZR* exon 10; *ROS1* exon 34 |
| TPM3-ROS1 | *TPM3* exon 8; *ROS1* exon 35 |
| GOPC-ROS1 | *GOPC* exon 4; *ROS1* exon 36 |
|  | *GOPC* exon 8; *ROS1* exon 35 |
| LRIG3-ROS1 | *LRIG3* exon 16; *ROS1* exon 35 |
| ***RET* fusions (7)** | |
| KIF5B-*RET* | *KIF5B* exon 15; *RET* exon 11 |
|  | *KIF5B* exon 15; *RET* exon 12 |
|  | *KIF5B* exon 16; *RET* exon 12 |
|  | *KIF5B* exon 22; *RET* exon 12 |
|  | *KIF5B* exon 23; *RET* exon 12 |
|  | *KIF5B* exon 24; *RET* exon 11 |
| CCDC6-*RET* | *CCDC6* exon 1; *RET* exon 12 |
| ***MET* exon 14 skipping** | |
| *MET* exon 14 skipping transcript detection at the exon 13 – exon 15 junction | |

Title: Multicenter evaluation of an automated, multiplex, RNA-based molecular assay for detection of ALK, ROS1, RET fusions and MET exon 14 skipping in NSCLC

**Virchows Archiv**

Authors: Melchior Linea, Hirschmann Astrid, Hofman Paul, Bontoux Christophe, Concha Angel, Mrabet-Dahbi Salima, Vannuffel Pascal, Watkin Emmanuel, Putzová Martina, Scarpino Stefania, Cayre Anne, Martin Paloma, Stoehr Robert, Hartmann Arndt

Corresponding author: Melchior Linea, Blegdamsvej 9, 2100 Ø København, +45 35455462, linea.cecilie.melchior@regionh.dk
