# Supplementary material for: Multicenter evaluation of an automated, multiplex, RNA-based molecular assay for detection of ALK, ROS1, RET fusions and MET exon 14 skipping in NSCLC
Source: Virchows Arch. 2024 Mar 16;484(4):677–86. doi: 10.1007/s00428-024-03778-9 (PMC11062995; doi:10.1007/s00428-024-03778-9)
Supplement: Supplementary file 2 — Supplementary Figure 1: Sample 19 Archer NGS routine reference result. (DOCX 1900 kb) [file 428_2024_3778_MOESM2_ESM.docx]

Supplementary Figure 1: Sample 19 Archer NGS routine reference result.

**A**


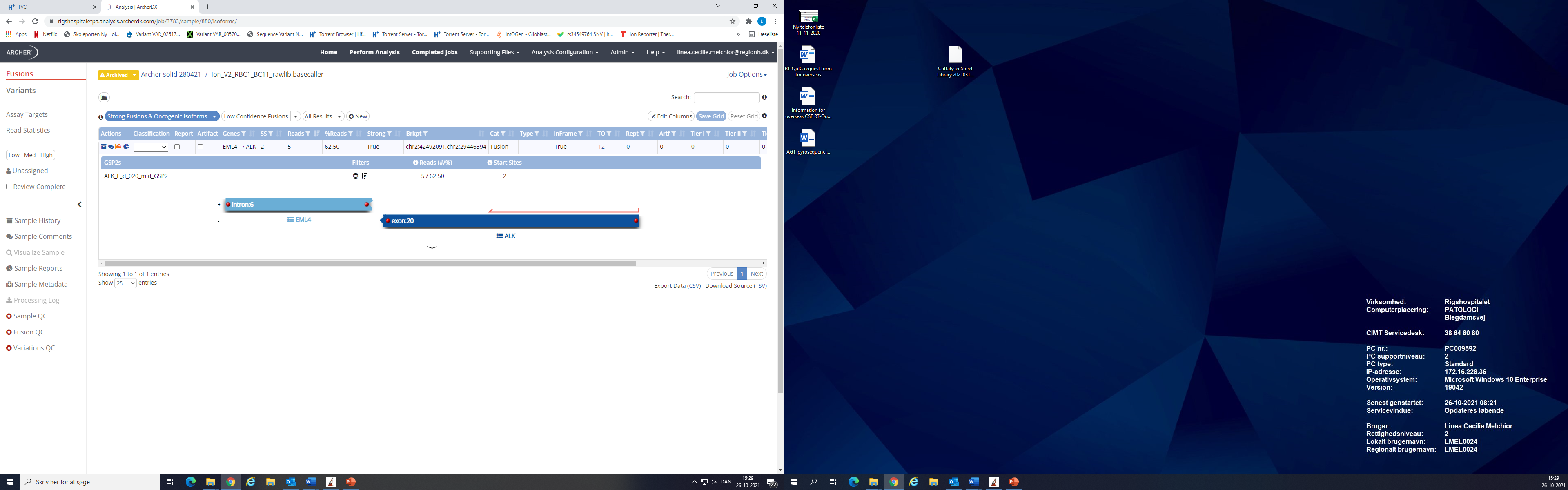


**B**


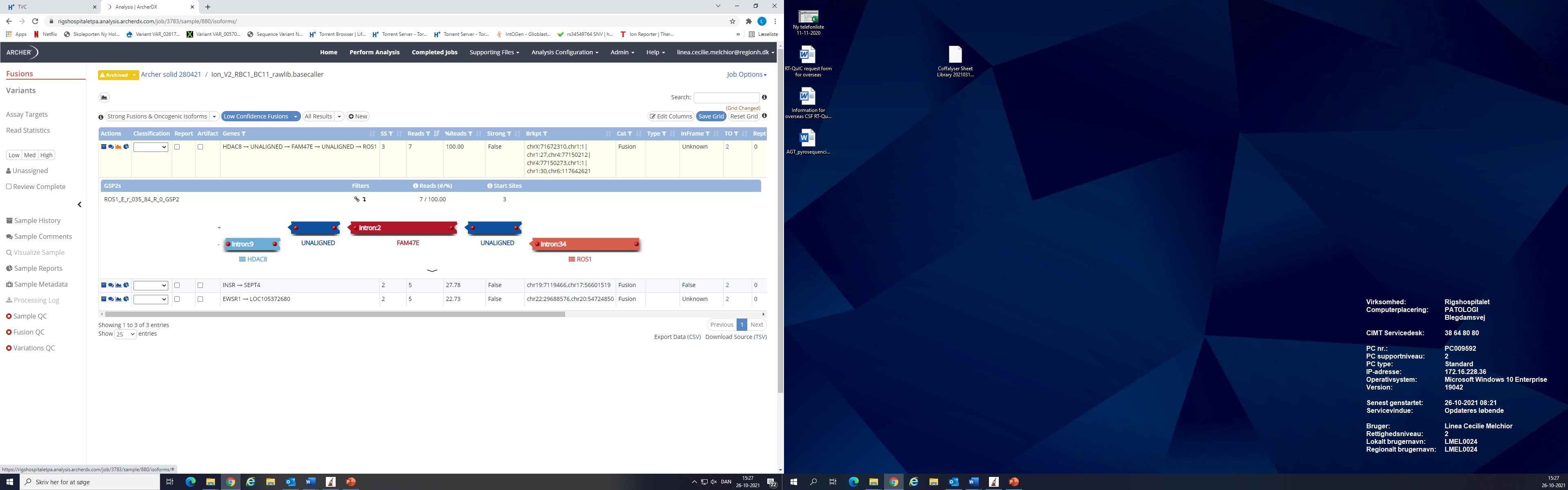


(A) *ALK* fusion NGS analysis result with positive call. (B) *ROS1* fusion NGS analysis result with negative call.

Title: Multicenter evaluation of an automated, multiplex, RNA-based molecular assay for detection of ALK, ROS1, RET fusions and MET exon 14 skipping in NSCLC

**Virchows Archiv**

Authors: Melchior Linea, Hirschmann Astrid, Hofman Paul, Bontoux Christophe, Concha Angel, Mrabet-Dahbi Salima, Vannuffel Pascal, Watkin Emmanuel, Putzová Martina, Scarpino Stefania, Cayre Anne, Martin Paloma, Stoehr Robert, Hartmann Arndt

Corresponding author: Melchior Linea, Blegdamsvej 9, 2100 Ø København, +45 35455462, linea.cecilie.melchior@regionh.dk
